# A Case of Complex Regional Pain Syndrome With Hypermobile Ehlers-Danlos Syndrome and Mast Cell Activation Syndrome: The Role of Unconventional Therapies

**DOI:** 10.7759/cureus.87898

**Published:** 2025-07-14

**Authors:** Zian Shabbir, Layla Mazdeyasnan, Mary McLain

**Affiliations:** 1 Research, California Health Sciences University - College of Osteopathic Medicine, Clovis, USA; 2 Central Valley Indian Health, Community Medical Center, Fresno, USA

**Keywords:** complex regional pain syndrome (crps), cromolyn sodium, ehlers-danlos syndrome, mast cell activation, montelukast

## Abstract

Complex regional pain syndrome (CRPS) type 2 is a chronic pain condition that develops after a nerve injury and is characterized by severe pain, allodynia, and functional impairments. Ehlers-Danlos syndrome hypermobility type (hEDS) and mast cell activation syndrome (MCAS) are connective tissue and inflammatory disorders that may contribute to the development of CRPS. Despite various treatment approaches, effective pain management remains a challenge, particularly in complex cases involving underlying genetic predispositions. We report the case of a 42-year-old female with a history of CRPS type 2, who was recently diagnosed with hEDS and had a family history of MCAS. Her clinical symptoms of chronic pain and gastrointestinal (GI) disturbances were unresponsive to typical therapeutic interventions. She was being managed with multiple medications. With this thought in mind, montelukast, a leukotriene receptor antagonist, was introduced, and the patient reported an initial improvement in pain. Cromolyn sodium, a mast cell stabilizer, was also added to her treatment regimen to further target her pain exacerbation. Finally, an anti-inflammatory focused GI regimen including betaine HCl, quercetin with bromelain, and other digestive enzymes was trialed. Her progress was monitored using a pain scale over the next six months. However, due to declining health, a subjective narrative scoring system replaced the Visual Analog Scale to represent her fluctuating and multifaceted symptom experience better. The relationship between hEDS, MCAS, and CRPS suggests a multifactorial pathogenesis involving connective tissue fragility, mast cell dysregulation, and neurogenic inflammation. Montelukast, cromolyn sodium, and GI supplementation represent potential therapeutic interventions for managing patients with CRPS linked to MCAS. These treatments offer a novel approach by targeting mast cell-mediated inflammation. This case emphasizes the need for further research into the role of mast cell stabilization in CRPS treatment to improve patient outcomes.

## Introduction

Complex regional pain syndrome (CRPS) is a chronic pain condition, typically occurring after injury or trauma. This disease is characterized by intense, disproportionate pain, swelling, and changes in the skin. CRPS type 2 (formerly known as causalgia, or burning pain) is associated with nerve injury and can lead to debilitating pain and significant functional impairment [1]. The pathophysiology of CRPS is multifactorial, involving dysregulation of both the central and peripheral nervous systems. However, in some cases, it may be due to an overactive inflammatory response [1]. CRPS has an estimated incidence of 5.5 to 26.2 cases per 100,000 person-years, with type 2 being the less common but more severe form.

In addition to its hallmark features of localized neuropathic pain, allodynia, and edema, CRPS may present with a broad spectrum of systemic complications involving the gastrointestinal (GI), endocrine, immune, and autonomic systems [2]. These symptoms arise from a combination of sympathetic nervous system dysregulation, chronic inflammation, and central sensitization. The thalamus, a central nervous system organizing hub, is responsible for the increased responsiveness to generally benign signals, such as touch and temperature, leading to inappropriately strong pain sensations known as allodynia. GI disturbances, including nausea, vomiting, constipation, and diarrhea, are also frequently reported but often underrecognized. These manifestations are thought to stem from autonomic imbalance and inflammatory mediator involvement. This is seen in patients with coexisting conditions such as mast cell activation syndrome (MCAS), which may amplify histamine-related gut symptoms [2].

In recent years, increasing evidence has highlighted the potential connection between connective tissue disorders, MCAS, and CRPS. One connective tissue disorder, Ehlers-Danlos syndrome hypermobility type (hEDS), is characterized by joint hypermobility, skin hyperextensibility, and tissue fragility [3]. hEDS affects an estimated 1 in 5,000 to 20,000 individuals, though underdiagnosis is likely due to variability in clinical presentation. Patients with hEDS often report chronic pain, frequent joint dislocations, and a heightened sensitivity to pain, which may predispose them to the development of CRPS [4]. The thought behind this association is due to defective collagen. These repeated microtraumas can lead to chronic nociceptive input, triggering central sensitization implicated in CRPS pathogenesis. Additionally, studies have shown that MCAS, a condition in which mast cells are inappropriately activated, releases mediators such as histamine. This etiology may contribute to inflammation and pain exacerbations in patients with CRPS [5,6].

In further comparison to existing literature, this case aligns with growing evidence that supports a potential pathophysiological triad between CRPS, hEDS, and MCAS. For instance, Weinstock et al. described the increased prevalence of neuropsychiatric and GI dysfunction in MCAS [6]. Although CRPS can be classified as neuropsychiatric in nature, no direct link is made here. Furthermore, Wasim et al. have demonstrated how the connective tissue fragility seen in hEDS may predispose patients to altered pain processing and autonomic dysregulation, both of which are prominent features in CRPS [7]. As for treatment strategies surrounding this, Li et al. attempted substance P-mediated control of MCAS in rats as a model for CRPS but yielded inconsistent results [8]. Of note, the findings from animal research may not always apply to humans. To our knowledge, no study has utilized mast cell-stabilizing medications and enzyme supplementation as a means of controlling CRPS-related symptoms.

The Visual Analog Scale (VAS) is a widely used, validated tool for assessing pain intensity. It typically consists of a 10 cm line ranging from “no pain” to “worst pain imaginable,” on which patients mark their pain level [9]. While effective in many acute and chronic pain conditions, the VAS is inherently limited in its ability to capture the qualitative, multifactorial, and fluctuating nature of pain syndromes such as CRPS. As our patient’s symptoms progressed, the VAS failed to adequately reflect the complexity of her pain, particularly when it involved GI distress, neuroinflammatory flares, and emotional exhaustion. Consequently, we transitioned to a subjective, narrative-based pain scoring system that allowed the patient to describe her pain contextually and holistically rather than through a number. Information regarding treatment was collected in interview-style follow-up appointments at three and six months after initiation of medication. Direct statements from the patient are included. This approach provided a more meaningful assessment of her symptom burden.

## Case presentation

History of presenting illness

**Figure 1:**
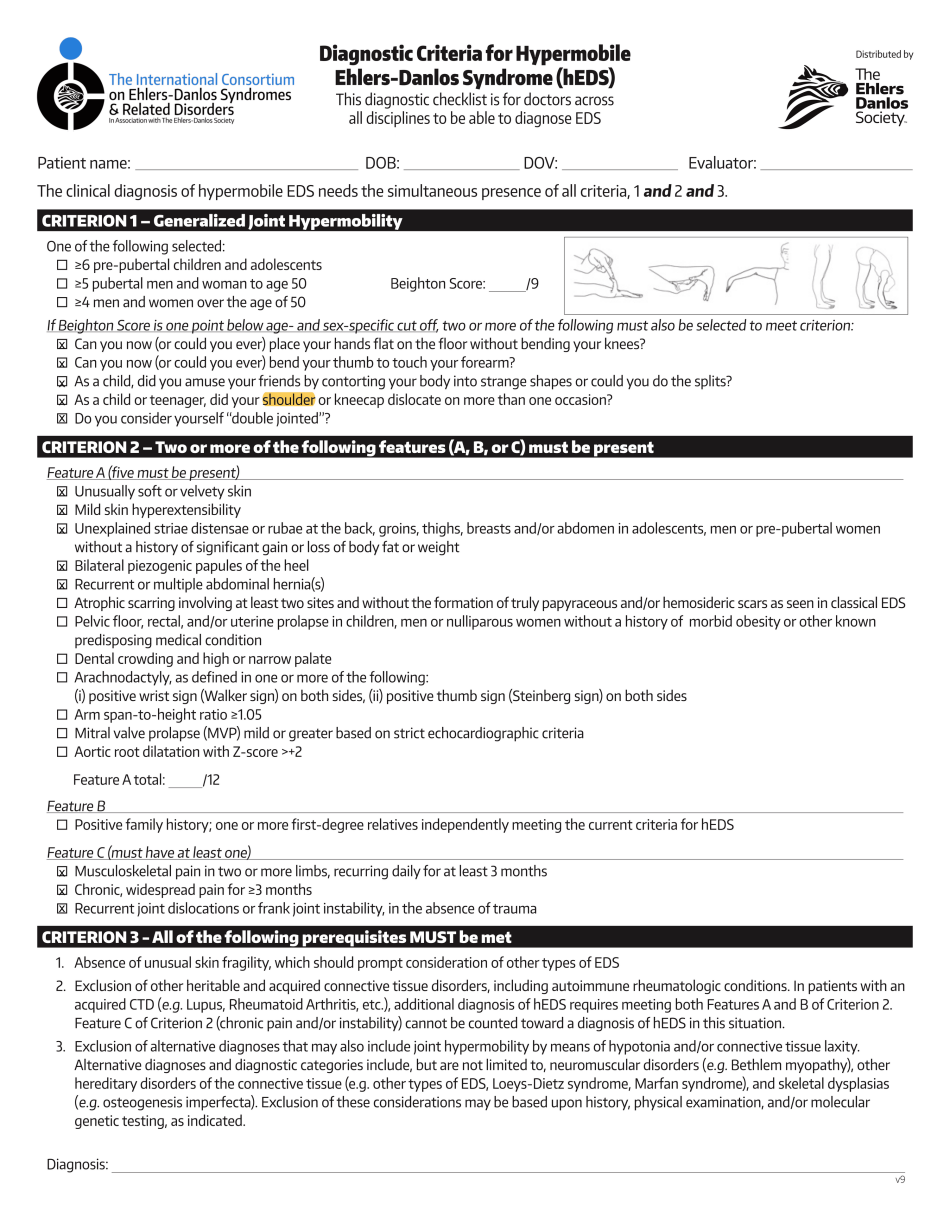
Beighton Score of the patient’s symptoms.

A 42-year-old female with a history of depression, anxiety, and back pain presented to her primary care physician with widespread pain and impaired mobility persisting for years. CRPS is a diagnosis of exclusion, and, as such, she was clinically diagnosed with CRPS type 2 due to a negative workup of other causes. Following a shoulder dislocation as a teenager, the patient recalled previous episodes of joint laxity, excessive flexibility, and bilateral pyogenic papules of the heel, among other diagnostic criteria for hEDS. She was then diagnosed with hEDS, using the Beighton score checklist in Figure 1 [10,11].

Family history

**Figure 2:**
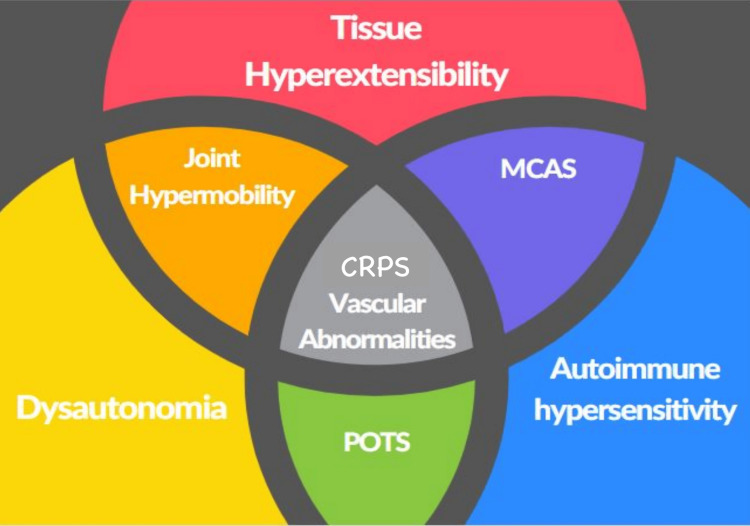
Intersect of various related conditions. MCAS = mast cell activation syndrome; CRPS = chronic regional pain syndrome; POTS = postural orthostatic tachycardia syndrome Reproduced from [12].

When gathering information about her family history, both her mother and sister had been diagnosed with MCAS, raising suspicion that she may also have an underlying MCAS disorder that was contributing to her CRPS and hypermobility symptoms. The patient agreed to undergo genetic testing for MCAS as part of her ongoing evaluation. In subsequent follow-up visits, the test results revealed she also tested positive. More specifically, for an increased number of *TSBAP1* gene copies, which may lead to increased tryptase activity by mast cells. According to the 2021 consensus criteria, MCAS diagnosis requires: (1) episodic symptoms consistent with mast cell mediator release in at least two systems (e.g., dermatologic, GI, cardiovascular); (2) an increase in serum tryptase during symptoms or other validated mast cell mediators; and (3) a documented clinical response to mast cell-targeted therapy. An overlap of symptoms and requirements, highlighted in Figure 2, may suggest that MCAS plays a role in both the onset and persistence of CRPS symptoms, especially in patients with underlying connective tissue disorders such as hEDS [6,12].

Medications

Her current medication regimen at this time included baclofen 20 mg three times a day, duloxetine HCl 30 mg once daily, gabapentin 600 mg three times a day, hydrocodone-acetaminophen 10-325 mg four times a day, ibuprofen 800 mg three times a day, lidocaine 5% patch three times a day, lorazepam 0.5 mg twice a day, ondansetron 4 mg two tablets twice a day, and propranolol HCl 10 mg two tablets three times a day. New medications trialed included montelukast sodium 10 mg once a day, cromolyn sodium nebulization solution 20 mg/2 mL inhaled as needed up to three times a day, and a variety of GI supplements as needed. These GI supplements included betaine HCl, quercetin with bromelain, and digestive enzymes. The plan to initiate these in this patient aimed to further reduce the frequency and severity of her pain flare-ups by targeting the root cause of mast cell dysregulation. Together, these medications offered a targeted approach to managing the complex interplay between MCAS and CRPS, addressing both the systemic inflammation and localized pain symptoms commonly seen in these conditions [13]. A complete list of medications can be found in Table 1.

**Table 1:** Current medication and supplement regimen. NSAID = non-steroidal anti-inflammatory drug; GABA = gamma amino-butyric acid; SNRI = serotonin-norepinephrine re-uptake inhibitor; TCA = tricyclic antidepressant

Category	Medication/Supplement	Dosage/Frequency	Mechanism of action	Role in this patient
Pain management	Hydrocodone-acetaminophen	10–325 mg four times daily	Opioid analgesic and antipyretic combination	Manages moderate-to-severe chronic pain, part of the baseline pain control regimen
Pain management	Ibuprofen	800 mg three times daily	NSAID; COX-1 and COX-2 inhibitor	Reduces inflammation and mild-to-moderate pain, supports opioid-sparing
Pain management	Baclofen	20 mg three times daily	GABA-B receptor agonist; muscle relaxant	Relieves muscle spasticity and reduces neuropathic pain tone
Pain management	Lidocaine patch	5%, applied three times daily	Sodium channel blocker (local anesthetic)	Provides localized analgesia for peripheral neuropathic pain
Neuropathic pain	Gabapentin	600 mg three times daily	Alpha-2-delta subunit calcium channel modulator	Treats neuropathic pain by dampening nerve excitability
Neuropathic pain	Duloxetine	30 mg once daily	SNRI; serotonin and norepinephrine reuptake inhibitor	Manages chronic pain and comorbid depression/anxiety
Neuropathic pain	Amitriptyline	100 mg once daily	TCA; inhibits serotonin/norepinephrine reuptake	Adjuvant for chronic pain and sleep disturbances
Inflammation/Mast cell stabilization	Montelukast	10 mg once daily	Leukotriene receptor antagonist	Reduces mast cell-mediated inflammation, partially improves symptoms
Inflammation/Mast cell stabilization	Cromolyn sodium	20 mg/2 mL via nebulizer up to three times a day	Mast cell stabilizer prevents mediator release	Tried for MCAS-related exacerbation, but paused due to the limited efficacy of total symptom control
Autonomic regulation	Propranolol HCl	10 mg two tablets three times daily	Non-selective beta-blocker	Helps control autonomic dysregulation symptoms (e.g., tachycardia, flushing)
Gastrointestinal/Nausea	Ondansetron	4 mg two tablets twice daily	5-HT3 receptor antagonist	Used for nausea associated with chronic illness and medications
Gastrointestinal/Nausea	Betaine HCl	1 capsule (750 mg) with meals	Provides hydrochloric acid to the stomach	Resolves diarrhea, improves digestion by compensating for hypochlorhydria
Gastrointestinal/Nausea	Digestive enzymes	1 capsule with meals	Pancreatic and brush border enzyme supplement	Improves nutrient absorption, reduces postprandial gastrointestinal pain
Gastrointestinal/Nausea	Gluten digestion pill	1 capsule with meals	DPP-IV enzyme-based gluten peptide breakdown	May prevent gluten-induced gut inflammation in sensitive individuals
Gastrointestinal/Nausea	Quercetin with bromelain	1 capsule (500 mg) between meals	Flavonoid with mast cell stabilization + proteolytic enzyme	Reduces histamine symptoms and inflammation, supports digestion
Psychiatric support	Lorazepam	0.5 mg twice daily	Benzodiazepine; GABA-A receptor modulator	Manages anxiety and situational distress related to pain burden

Montelukast

Montelukast is a leukotriene receptor antagonist that primarily functions by blocking the effects of leukotrienes, which are inflammatory mediators released by mast cells [14]. In patients with MCAS, these mediators are excessively produced, leading to inflammation, pain, and hypersensitivity responses [13]. By inhibiting leukotriene activity, montelukast reduces mast cell-driven inflammation, which may alleviate symptoms of CRPS, such as pain and swelling. Studies suggest that montelukast’s anti-inflammatory effects can contribute to improved pain control in CRPS, particularly in patients with coexisting MCAS [14]. During her six-month follow-up with her primary care physician, the patient reported “notable improvement” in her pain symptoms following the introduction of montelukast. She claimed it has “helped her pain overall” and will continue it as a mainstay therapy. The patient’s improvement in general pain symptoms after starting montelukast further supports its potential role in managing the inflammatory aspect of CRPS.

Cromolyn Sodium

Cromolyn sodium is a mast cell stabilizer that works by preventing the release of histamine and other pro-inflammatory mediators from mast cells [15]. This drug is effective in reducing systemic mast cell activation and may help mitigate the excessive inflammatory response seen in both MCAS and CRPS [15]. By stabilizing mast cells and reducing mediator release, cromolyn sodium offers a new therapeutic approach to controlling pain and hypersensitivity associated with CRPS. At her six-month follow-up, the patient was started on cromolyn sodium nebulization Solution 20 mg/2 mL as needed up to three times a day. This drug acts as a potential therapeutic intervention for CRPS symptoms. During her appointment, the patient reported that the cromolyn “did not help as much as the GI supplements.” The patient was using cromolyn “for a few months, including it with [her] meditation breathing techniques several times a day. [She] did not see very much difference at that time but will start it up again, now that her diarrhea has stopped, to see if she can notice a difference.” As such, more time is needed to uncover the true potential of cromolyn in symptom management.

GI Supplementation

The patient’s GI symptoms had become a significant component of her overall disease burden, contributing to marked weight loss and functional decline. She reported severe postprandial pain, early satiety, and diarrhea, which had collectively led to a greater than 25% reduction in her body weight. These symptoms are likely multifactorial but are suspected to be driven by mast cell dysregulation, inflammation, and autonomic dysfunction [6]. Notably, traditional antihistamines exacerbated her symptoms, further supporting a non-traditional or paradoxical histamine response common in some MCAS cases [5,6].

To address her GI issues, the patient began using betaine HCl to increase gastric acidity. Betaine HCl has been shown to benefit individuals with hypochlorhydria by promoting protein digestion and nutrient absorption and by reducing GI bacterial overgrowth [16]. After initiating this therapy, the patient claimed her “diarrhea stopped after taking Betaine HCl. That was a lifesaver.” She experienced near-complete resolution of diarrhea, suggesting that low stomach acid had been contributing to her dysmotility and malabsorption. The symptoms improved notably following the use of betaine HCl.

To further reduce histamine-related inflammation, the patient initiated a regimen of quercetin with bromelain. Quercetin has demonstrated the ability to stabilize mast cells and inhibit histamine release. In addition, Bromelain improves quercetin’s absorption and offers additional anti-inflammatory effects [17,18]. Together, these therapies provided “symptom relief and improved [her] tolerance to food.”

The patient also began taking broad-spectrum digestive enzymes, which assist in the enzymatic breakdown of proteins, carbohydrates, and fats. These enzymes reduce the metabolic strain on the GI tract and have been shown to improve nutrient absorption and decrease symptoms such as bloating and discomfort [19]. She also used a gluten digestion aid containing DPP-IV enzymes, which has been explored as a supplement for gluten-sensitive individuals [20]. This GI supplement regimen has become a vital component in the patient’s care plan. It highlights the underrecognized GI manifestations of CRPS and MCAS and the need for individualized, non-pharmacological strategies in managing complex pain syndromes.

## Discussion

This case underscores the clinical complexity of managing CRPS in a patient with coexisting hEDS and confirmed MCAS. The overlapping pathophysiology of these conditions contributes to the severity and refractoriness of her symptoms. CRPS is classically described as a localized neuropathic disorder, but this case illustrates its potential for widespread systemic involvement [2]. In this patient, GI symptoms debilitated and exacerbated her overall disease burden, contributing to significant weight loss and nutritional compromise.

The patient’s lack of response to conventional antihistamines and the paradoxical worsening of symptoms point toward atypical mast cell behavior, a hallmark feature of MCAS [5,6]. While montelukast offered consistent improvement in her pain levels and was well-tolerated, cromolyn sodium provided minimal perceived benefit and was eventually paused. These outcomes reflect the variable efficacy of mast cell-targeted therapies and highlight the need for individualized patient care. Notably, the patient experienced meaningful symptom relief with integrative GI-focused interventions, including betaine HCl, quercetin with bromelain, and digestive enzymes. These supplements likely contributed to improved digestion, reduced mast cell activity, and better tolerance of food, demonstrating the value of a non-pharmacologic approach.

This report brings attention to the limitations of traditional pain assessment tools in complex, multisystem disorders. The transition from the VAS to a subjective narrative pain scale allowed for a more comprehensive and personalized understanding of the patient’s fluctuating symptoms. It captured pain intensity and the impact of pain on her emotional state, functionality, and daily living factors. However, the use of this subjective pain reporting limits generalizability.

With a confirmed CRPS diagnosis, the patient is now proceeding with SCS implantation. This represents a key escalation in her treatment plan and is typically considered when conservative medical management fails to yield sufficient pain relief. SCS is a neuromodulatory intervention that delivers electrical impulses to the dorsal columns of the spinal cord, aiming to disrupt aberrant pain signaling pathways and reduce central sensitization. In clinical practice, SCS is often viewed as a later-stage or “last resort” therapy, reserved for patients with refractory symptoms who have exhausted standard pharmacologic and rehabilitative approaches. The decision to proceed with SCS reflects a shared decision-making process grounded in the patient’s treatment fatigue, diminished quality of life, and her prior incomplete response to conventional and integrative therapies. As she continues her multidisciplinary care, SCS may serve as a stabilizing component of an individualized pain management strategy.

Several important limitations must be acknowledged. Most notably, the assessment of treatment efficacy in this case was based entirely on subjective reporting. While the VAS was initially considered, the patient was unable to reliably quantify her pain due to the complex multisystemic nature of her symptoms. Consequently, a narrative-based, qualitative pain assessment was adopted, in which symptom improvement or worsening was described directly by the patient in her own words. Although this approach provided valuable clinical insight, it lacks the objectivity and reproducibility of standardized analytical measures.

Additionally, multiple therapeutic interventions were introduced in overlapping timeframes without standardized washout periods. This polytherapeutic approach limits the ability to isolate the effects of any single intervention and introduces confounding variables that undermine the strength of any causal inference. This limitation reflects a violation of core clinical research principles regarding intervention attribution and temporal association.

The continuation of pre-existing medications and supplements during the trial period further complicates the interpretation of the treatment outcomes. As such, the findings in this report should be interpreted as observational and hypothesis-generating rather than confirmatory. The intent of this case is not to establish statistical significance but to explore a clinically relevant phenomenon that may warrant further investigation. The inferences that can be gathered from this report set the framework for future statistically relevant and eventually clinically reproducible outcomes.

For example, future studies with a prospective design, standardized symptom tracking tools, and controlled intervention protocols are essential to validate the therapeutic potential of mast cell stabilizers and GI modulators in CRPS. Such research should aim to disentangle the complex interplay between the nervous, immune, and connective tissue systems in CRPS and comorbid syndromes such as hEDS and MCAS.

Despite its limitations, this case highlights an underrecognized intersection of conditions and introduces a potentially novel symptom management strategy, offering a foundation for future research into more targeted therapies for refractory CRPS. Nonetheless, the coexistence of hEDS, MCAS, and CRPS suggests possible interactions, though mechanistic evidence remains hypothetical.

## Conclusions

The management of CRPS necessitates continuous monitoring and individualized treatment adjustments due to the dynamic nature of symptoms and the risk of central and peripheral sensitization. This case highlights the critical need for a coordinated, multidisciplinary approach to care, especially when patients present with overlapping syndromes such as hEDS and MCAS that may amplify or obscure CRPS symptomatology.
